# Looking at faces in the wild

**DOI:** 10.1038/s41598-022-25268-1

**Published:** 2023-01-16

**Authors:** Victor P. L. Varela, Alice Towler, Richard I. Kemp, David White

**Affiliations:** 1 University of New South Wales, Sydney, Australia; 2 University of Queensland, Brisbane, Australia

**Keywords:** Psychology, Human behaviour

## Abstract

Faces are key to everyday social interactions, but our understanding of social attention is based on experiments that present images of faces on computer screens. Advances in wearable eye-tracking devices now enable studies in unconstrained natural settings but this approach has been limited by manual coding of fixations. Here we introduce an automatic ‘dynamic region of interest’ approach that registers eye-fixations to bodies and faces seen while a participant moves through the environment. We show that just 14% of fixations are to faces of passersby, contrasting with prior screen-based studies that suggest faces automatically capture visual attention. We also demonstrate the potential for this new tool to help understand differences in individuals’ social attention, and the content of their perceptual exposure to other people. Together, this can form the basis of a new paradigm for studying social attention ‘in the wild’ that opens new avenues for theoretical, applied and clinical research.

A brief glance at a person signals a wealth of critical social information. Information about their emotional state, intentions, demographics and identity all serve to enable us to navigate our social world successfully. Understanding the perceptual processes responsible for these impressive abilities has been an important focus of research in social cognition.

One approach to studying person perception is to examine socially-directed attention by analysing people's eye movements as they view images of people presented on computer screens (e.g.^1–6^). However, photographs of social scenes do not represent the dynamic and multidimensional reality of our social experience. Indeed, participants fixate on faces less in face-to-face interactions than when watching video stimuli^7–10^, indicating that contrived laboratory tasks are inadequate analogues of real-world social attention (^11^ see also^7,8^).

Surprisingly little is known about how people direct their attention towards others in natural settings. Yet, this information provides valuable constraints to understanding the perceptual processes and mechanisms of attention. For example, researchers have captured the visual experience of babies and toddlers using wearable cameras, enabling researchers to investgate how perceptual expertise with faces develops. This work shows that faces are present in infants’ field of view roughly 25% of the time (e.g.^12^), with the vast majority of this exposure being to familiar faces of primary caregivers. In contrast, faces make up a far smaller fraction of children’s visual experience beyond their first birthday (~ 10%, e.g.^13,14^). The extent to which babies and children *attend* to these faces is less clear. More generally, quantifying and characterising faces that are attended provides the basis for developing theories of perceptual expertise, by grounding them in the visual information sampled from the environment (e.g. see^15^).

Studies of adults’ attention to people in natural settings are extremely rare, and almost all knowledge on this topic comes from tightly controlled laboratory-based research. This laboratory-based research shows, for example, that faces capture attention and are processed preferentially relative to non-face objects and bodies^16–18^, and this leads to the view that this process is automatic (^19^, for a review see^20^). However, it is not clear whether this holds for ambient environments populated with many competing stimuli each with its unique affordance^21^ and where the ‘social stimuli’ are real people, complete with minds, eyes and legs of their own.

This knowledge gap has increased interest in methods that allow studies of person perception and social attention in immersive environments. One approach has been to use virtual reality, with faces rendered on animated bodies in virtual worlds^22–24^. Another has been to study social attention in “the wild” by studying the eyemovements of participants wearing eye-tracking devices that monitor their fixations as they navigate real-world ambient environments (for recent reviews see^10,25,26^.

Wearable eye-tracking offers the advantage of studying social attention and person perception in situ. However, it requires experimenters to manually code what is being fixated on every fixation, amounting to thousands of manual coding decisions even for a single participant in a short 10 min study. Even coding simple aspects of gaze fixations, for example, counting person fixations vs non-person fixations, is extremely time-consuming^25,27,28^. It is therefore impractical to examine social attention in naturalistic settings at the resolution afforded by lab-based eye-tracking studies (e.g.^1,4,29^). Experimenters wishing to conduct naturalistic studies of social attention are therefore limited to costly, coarse analysis of fixation patterns.

Here we introduce a novel method that automates fine-grained investigations of naturalistic social attention for the first time. Our ‘dynamic regions of interest’ (dROI) approach automatically measures social attention in ambient environments frame-by-frame. We achieve this by co-registering eye-movement data from a wearable eye-tracker with body and face landmark positions extracted from video data using a state-of-the-art computer vision algorithm^30^. This encodes eye fixations directed towards people and maps fixations to landmarks on the face and body. A demonstration of the dROI method is available in the Supplementary Video.

Our approach overcomes many significant limitations of prior work on social attention in natural settings, saving substantial research effort by avoiding the need for manual coding of fixations to pre-specified regions (e.g.^27,28,31–33^). In addition to removing the burden of manual coding, our approach also increases temporal resolution and the volume of data, enabling new analytic approaches which open up new avenues to study person perception ‘in the wild’.

Given this is the first paper to use this approach, we address some preliminary research questions to demonstrate its potential for answering a diverse range of questions related to social attention. Our primary aim was to quantify the extent to which people attend to bodies and faces of passersby and ask whether faces ‘capture’ viewers’ attention as claimed on the basis of lab-based studies (e.g.^16–18^). We also conducted an exploratory analysis to examine whether patterns of social in natural settings may reflect stable individual differences in observers, both when participants were walking in a public space and when they were engaged in face-to-face social interaction.

## Results

### Faces of passersby do not capture attention in a live natural setting

**Figure 1:**
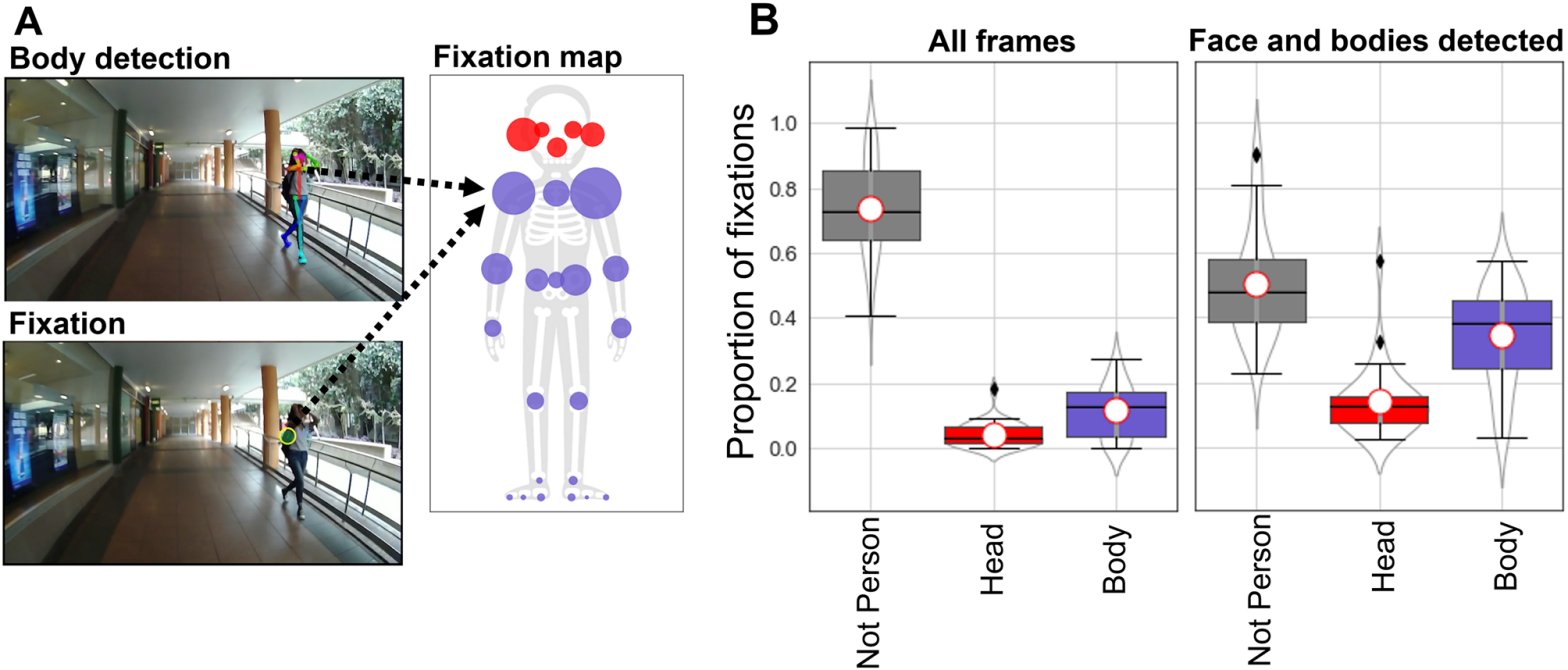
Dynamic region of interest (dROI) analysis of social attention while navigating a university campus. (**A**) Using data from a wearable eye-tracker, we extracted body landmarks from videos using OpenPose (top left) and co-registered viewers’ fixations towards these landmarks (bottom left). The skeleton figure shows a participant’s relative proportion of fixations to each body landmark, indicated by the size of the marker (all individual participant maps are available in Supplementary Materials, Sect. 1.4 and a video demonstration of the dROI method is shown in the Supplementary Video). (**B**) The left data panel shows boxplots of the proportions of non-person, head and body fixations as a proportion of all fixations in the recordings. The right data panel shows the proportions of non-person, head and body fixations only as a proportion of frames where the algorithm detected heads and bodies. See main text for analysis.

Thirty-three participants followed a circular route around a busy university campus wearing a mobile eye-tracking device (see ‘Methods—Data Collection’ for full technical and procedural details). We show an example video frame illustrating the eye-tracking data provided by the eye-tracker and the detected dynamic regions of interest in Fig. 1A (left panel). Our dynamic region of interest (dROI) analysis of social attention relied on automatic face and body detection algorithms developed by Cao and colleagues (OpenPose:^30^). We verified the accuracy of this algorithm on our video data by comparing its detections to manual coding of body presence by four human observers and found a high level of agreement (see Supplementary Materials Section 1.1; see also^34^).

To calculate the proportion of fixations participants made to faces and bodies, we co-registered fixation locations from the eye-tracker with landmarks on faces and bodies (Fig. 1A, see Methods—Eye gaze processing). The average width of heads detected in the scene measured 2.2° of visual angle from ear-to-ear which roughly corresponds with prior lab-based work showing attention capture by faces (e.g.^16,17^; see Supplementary Material Section. 1.2 for full head size data). For reference, the width of the head in Fig. 1A, as perceived by the participant, measured 4° visual angle from ear to ear, with a body height of 32° from chin to toe.

As a function of total fixations (Fig. 1B, left), 16% of fixations were directed to people, with just 4% directed at people’s heads (Body: M = 11.6%, SD = 8.3%; Head: M = 4.3%, SD = 3.8%). Restricting the analysis to only frames where faces and bodies were detected by the algorithm, we observed higher proportions of fixation towards people (50%), but fixations to heads remained relatively low at 14% of fixations (Body: M = 34.4%, SD = 14.9%; Head: M = 14.4%, SD = 10.3%). The small proportion of fixations to faces may suggest that the widely reported finding that ‘faces capture attention’ in lab-based studies does not transfer to encounters with unfamiliar people in public spaces (e.g.^4–6,16,17,35–37^).

Because there were large variations in face width for detected faces in the field of vision (M = 2.24°, SD = 1.28°,min = 0.13°, max = 14.36), we repeated this analysis separately for above- and below-average sized faces and found a modest increase in attention to above-average sized faces (15.2% v 19.1%). However the same pattern of results shown in Fig. 1B was observed (see Supplementary Material Section 1.3).

### The effect of frontal vs. averted faces on attention in live natural setting

**Figure 2:**
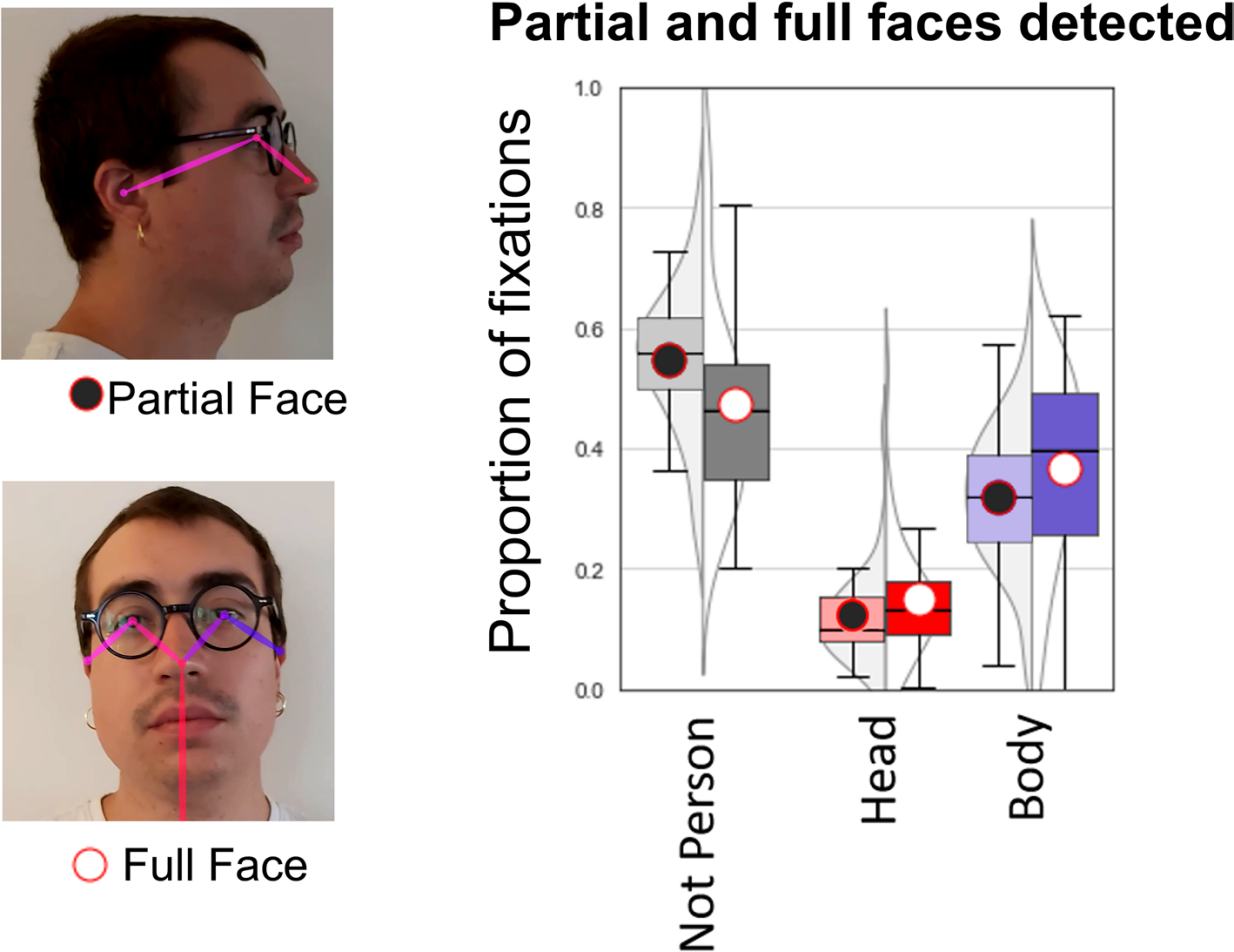
Comparing social attention to faces when they are fully visible versus partially visible in a video frame. Partially visible faces were due to averted head angle, or in some rare cases occlusion (top left). Results show a greater proportion of fixations to people when their faces were fully visible (see figure legend on left). See main text for analysis and Supplementary Materials (Sect. 2) for the full ANOVA and further details of the computational approach used to classify face type.

Previous lab-based studies have shown that frontal faces capture more attention than averted faces (e.g.^38,39^). In a further test of whether unfamiliar faces capture attention in naturalistic settings, we compared the proportions of fixations to people in frames where the algorithm detected full faces (i.e. all facial features) against when the algorithm detected partial faces (i.e. subset of facial features; see Fig. 2 left, and Supplementary Materials Section 2.1 for the manual process used to verify this approach). This provided a test of whether frontal faces are fixated more than averted faces in a natural setting.

Figure 2 shows that participants made more fixations to people with fully visible frontal faces relative to averted face. However, ANOVA simple main effects showed that this increase in attention was distributed evenly between head regions (Partial = 12.3%, Full = 15.1%: *F*(1,30) = 7.035, *p* = 0.013, *ç*) and body regions (Partial = 32.1%, Full = 36.6%: *F*(1,30) = 6.64, *p* < 0.015, *η*^*2*^_*p*_ = 0.181; see Supplementary Materials Section 2.2 for full ANOVA). This result suggests that participants were more likely to fixate on *people* when their faces were in full view, but provides no evidence that faces captured this attention any more than other body regions.

### Fixation patterns during face-to-face interaction

**Figure 3:**
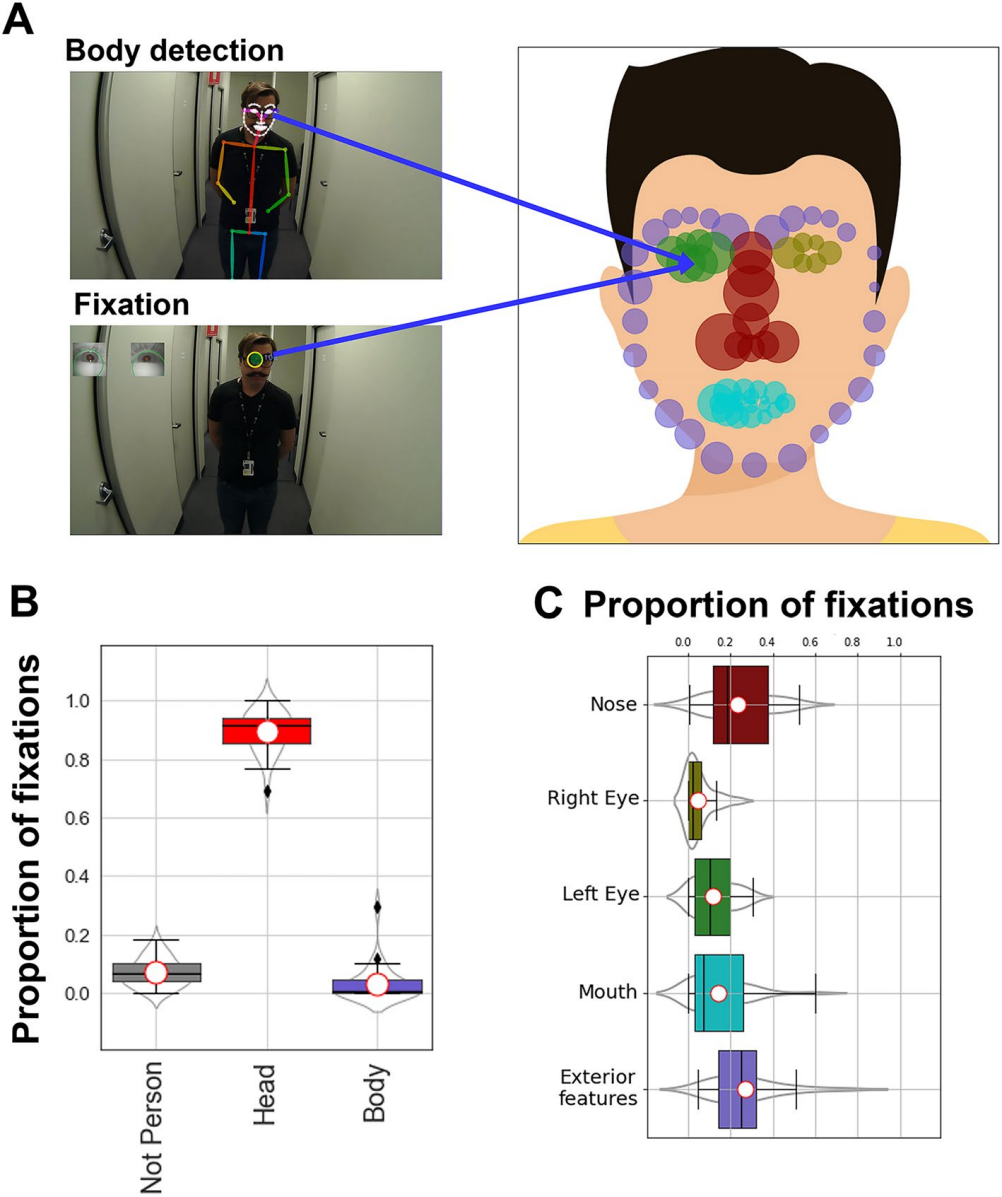
Dynamic region of interest analysis applied to face-to-face interaction. (**A**) We extracted facial landmarks from the video source using OpenPose (left), and these landmarks were used to register the viewers’ fixation positions on the face. The width of the head from ear to ear in this image corresponds to 12.6° of visual angle. The size of circles on the schematic face shows the average proportion of fixations participants made to each landmark (individual participant fixation maps are available in Supplementary Material Sect. 3.1); (**B**) Relative frequency of fixations to the experimenter’s face and body compared to the surrounding environment; (**C**) Relative frequency of fixations to facial regions indexed by colour coded mapping to landmarks shown in in panel A (right).

We also recorded participants’ fixation patterns during a face-to-face conversation with the experimenter (see Methods—Data collection). This conversation occurred before the main navigation task, as participants listened to scripted task instructions for about 30 s before asking any follow-up questions. Because participants were closer to the experimenter and faces were larger than in the navigation task (M = 8.17°, SD = 1.17°,min = 3.02°, max = 11.87°; see Supplementary Materials, Fig. S3), this enabled the face detection algorithm to detect 70 facial landmarks (See Methods—Eye gaze processing, Data Analysis Strategy). An example of the video recording and the proportion of fixations to each facial landmark for one participant is shown in Fig. 3A.

Unsurprisingly, participants spent far longer looking at faces when engaged in conversation compared to in the navigation task, with an average of 92.9% of fixations to people and 89.5% to faces (Fig. 3B). The proportion of fixations to different regions of the experimenter’s face is shown in Fig. 3C, showing a focus on internal facial features, in line with screen-based eye-tracking studies (e.g.^1,40,41^. However, these regions are computed by assigning fixations to the closest landmark, which lacks precision because landmarks are not distributed evenly across the face. To resolve this issue, we used the spatial relations between facial landmarks and fixations to triangulate precise fixation locations (see Methods—Eye gaze processing).

**Figure 4:**
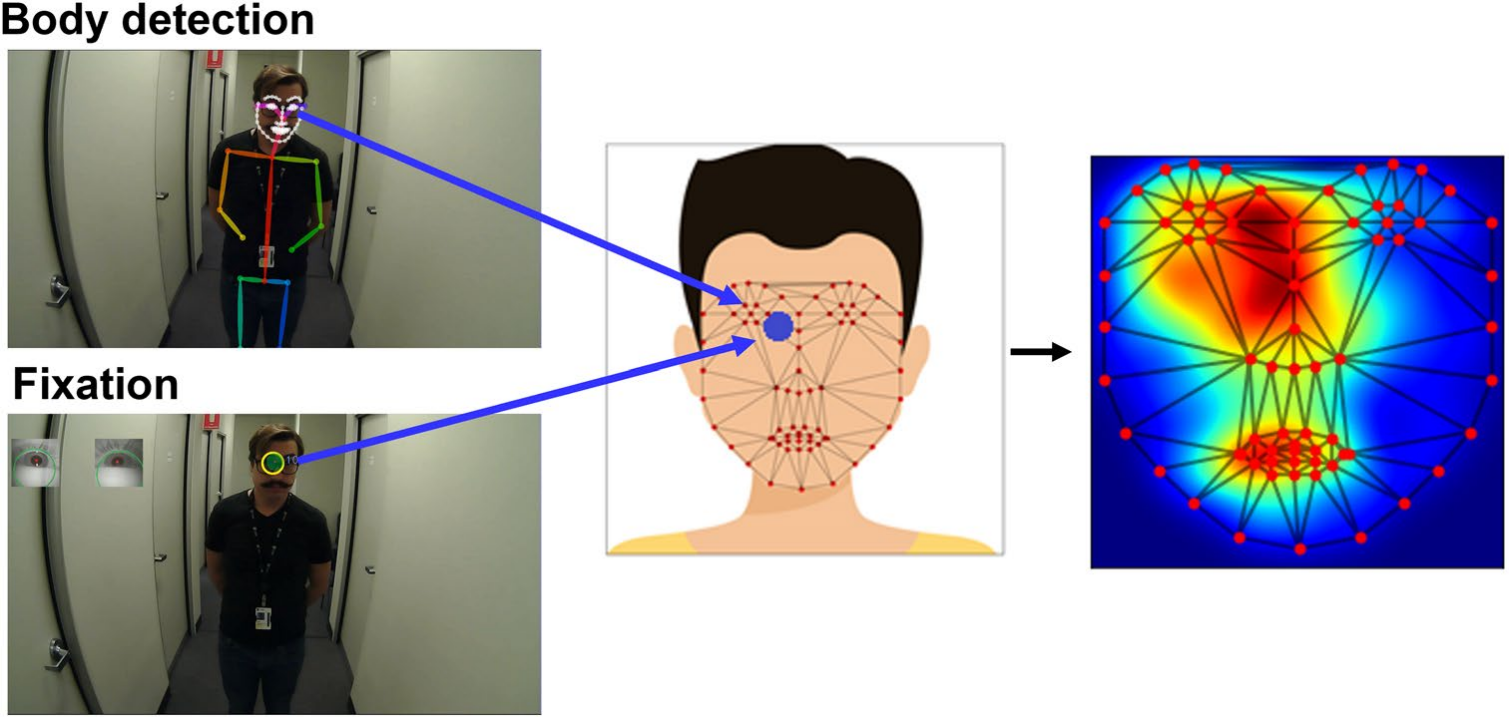
Heatmap analysis of face-to-face interaction. Video and eye movement from the wearable eye-tracker (left) were registered using OpenPose facial landmarks and converted to locations on the standard face template using Delaunay triangulation and affine transformations (middle). This technique enabled face fixation data to be aggregated and recorded as heatmaps (right; see Supplementary Materials Sect. 3.2 for individual participant heatmaps).

Triangulation enabled us to compute heatmaps of participants gaze patterns distributed continuously across the face, as shown in the average heatmap on Fig. 4 (see Supplementary Materials Section 3.2 for participant’s individual heatmaps). This average heatmap shows a tendency for participants to focus on the eyes, nose and mouth in a ‘T’ shaped distribution, which is a common finding in screen-based eye-tracking studies [e.g.^1,40,41^]). Interestingly, there is also a clear leftward bias observable in Fig. 4. This bias is consistent with previous laboratory-based research investigating people looking at faces on screens to perceive identity^42^ and detect emotional expression^43–45^.

### Individual differences in naturalistic social attention

Computerised lab-based tests have established that individual differences in social attention are stable across test sessions, and these differences are associated with genetic variation (e.g.^46,47^). Although it is not possible to make strong inferences about individual differences based on our relatively small sample size of 30 participants, we conducted a preliminary correlational analysis of social attention during the navigation task.

We first calculated the correlation between individuals’ tendency to fixate on people and faces in two distinct segments of the navigation study route that were separated by a short rest break (see Methods—Data collection). We found a significant correlation (Spearman's rho = 0.58, *p* = 0.001, CI = [0.27,0.78], n = 30; see scatterplot in Supplementary Materials, Fig. S10), indicating that individual differences in people’s tendency to fixate on people and faces was relatively stable across our test. Because the university campus was busier for some participants than others, participants’ proportion of fixations to people may be affected by number of people available in the scene. We therefore repeated the correlational analysis controlling for the average number of people per frame for each participant, and found the same pattern of stable individual differences (Spearman's rho = 0.532, *p* = 0.002, CI = [0.21,0.75], n = 30; see Supplementary Material Section 4.1 for further details of this analysis).

Participants in our study had completed measures of self-reported and objective face recognition ability (Cambridge Face Memory Test extended version^48^, Prosopagnosia Index short version^49^; see Methods—Data Collection). So we also conducted an exploratory analysis to examine if there was evidence of an association between attention to faces and people and face identity processing ability, but we found no significant association (see Supplementary Material Section 4.2 for further details). Exploratory analysis of individual gaze patterns to faces in the face-to-face interaction are also reported in Supplementary Materials Section 4.3 and show a modest but inconclusive association with measures of face identity processing ability.

### Capturing exposure to faces in the wild

Analysis in this paper is focused on quantifying viewers’ attention to people in their environment. However, the automated approach we have developed can also capture the *content* of person information sampled from these environments. In the field of face perception, the type of face information that is sampled from the environment has special theoretical significance, because exposure to faces is argued to underpin people’s specialised expertise in processing faces (e.g. see^50^). The concept of ‘face diet’ tends to refer to the fact that people tend to be exposed to faces that are from similar demographic groups to themselves, and this has been used to explain the ‘other-race effect’ whereby people are better at recognising faces of their own ethnicity (e.g.^51,52^). But the composition of face exposure varies on many more dimensions, including transient properties of the face including head angle, lighting conditions and expression. The influence of attention on naturalistic face diets is unknown.

**Figure 5:**
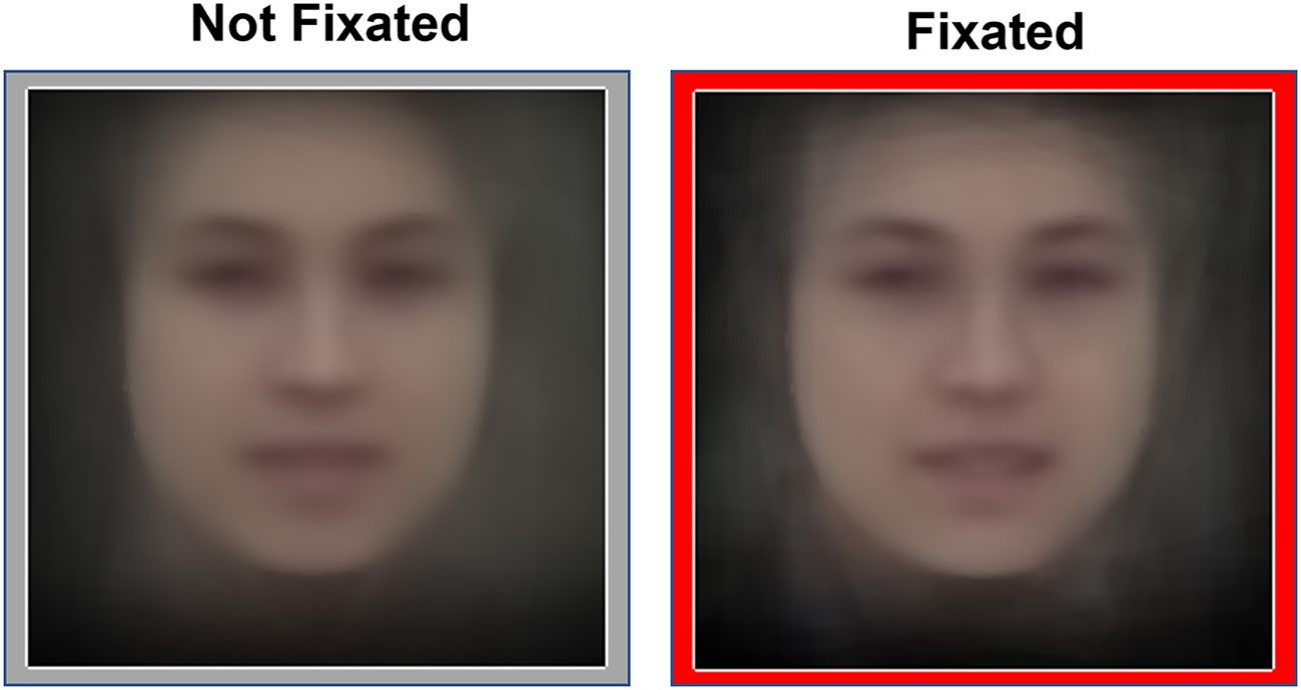
Image averages showing fixated and non-fixated views of the same faces. Average images of fully detected faces that were not fixated (left) and fixated (right) across all participants. This method is shown here for an illustration of what is achievable using our approach but is not intended as formal analysis. A map showing the locations of face images that contributed to these averages in the field of view is shown in Fig. S4 of Supplementary Material.

In Fig. 5, we demonstrate how combining automatic face detection with wearable eye-tracking can be used to explore the way that attention shapes ‘face diets’. We focus on differences in ‘within-face variation’ for fixated and non-fixated faces (i.e. differences in transient aspects of facial appearance such as systematic differences in lighting, head angle or expression). We limited this analysis to faces that were fully detected with 70 facial landmarks detected, to allow further image processing. For each face that was both fully detected and fixated, we extracted both the frames in which it was fixated (n = 1601) and the frames in which it was not fixated (n = 4754). We then generated image averages of fixated and non-fixated face images via an image morphing procedure using the 70 face landmark coordinates detected by OpenPose (see Methods—Data analysis).

We computed averages in Fig. 5 so that they contained the same contribution for each face identity in each average. This means that any differences between the images is due to differences in transient aspects of appearance, and there do appear to be subtle differences in expression and lighting. Due to the small number of faces that were fully detected in our navigation task, it was not possible to create averages for individual participants in our study, but studies with longer durations and/or higher resolution cameras may not face this same limitation. Therefore, although this preliminary work does not support inferences about systematic biases in face information sampling, it demonstrates future potential to understand the influence of attention on naturalistic face diets, and differences in demographic composition of this diet across different viewer demographics (e.g. see^53^).

## Discussion

Our primary research goal was to develop and validate a new research tool to study social attention ‘in the wild’. While automated registration of fixations to faces has been applied to static eye-tracking videos viewed on screen^54,55^, this is the first time they have been applied to wearable eye-trackers outside of the laboratory. Measures of fixation proportions to people and bodies in a natural setting were broadly consistent with prior research using manual coding of video recordings from wearable eye-trackers^27,28^. Further, there was high agreement between our automated measures and manual experimenter coding (see Fig. S7), and measures of individual participant’s fixation patterns were reliable over repeated measurements. Finally, in face-to-face interaction, patterns of fixations across face regions were consistent with general patterns observed in screen-based studies. Together, we interpret this as evidence that dynamic region of interest (dROI) approaches are valid for studying social attention in natural settings.

The dROI approach enabled us to ask some preliminary questions, inspired by screen-based studies of social attention, in natural settings for the first time. We first examined the extent to which faces automatically capture attention as participants navigated a busy public space. Contrary to conclusions based on lab-based experiments (e.g.^4–6,16,17,35–37^), we found no evidence that faces automatically capture attention ‘in the wild’. Fixations to faces—when faces were visible in participants' field of view—made up a small proportion of total fixations (14%). Moreover, when comparing attention capture by faces and bodies that were fully visible and those that were only partially viewable, we found that fully visible faces increased fixations to both faces and bodies equivalently. This evidence does not support the idea that people automatically orient their attention to faces, at least for unfamiliar faces in a public space.

As expected, we found that participants spent a far greater proportion of time looking at faces during one-to-one social interaction. This difference is consistent with fixation patterns to faces appearing on-screen, which are also highly context-dependent. For example, attention is dependent on task instructions^56,57^, whether the face is moving^58–60^, speaking^58,61^ and the non-verbal behaviour of the viewed person (^61^, for a review see^62^).

Screen-based eye-tracking with free viewing of static scenes—with no task or instruction—show around 60% of fixations to faces where people are prominent in the scene^4,57^ although other studies report lower estimates^56^. This variance between studies may be due to simple properties such as the prominence or size of faces in the scene, or to higher-order aspects of the images. The paradigm we have presented here provides a unique opportunity to disentangle how attention is influenced by the stimulus, situational context, as well as the goals and motivations of viewers (see^1,57,63,64^ in natural settings.

Our study also points to two additional directions for future research made possible by our dROI approach. First, it opens new possibilities for understanding individual differences in social attention and face-processing ability^65^. Individual differences in attention to people were stable across different sections of the navigation route, which is consistent with screen-based eye-tracking studies that show a strong hereditary influence on patterns of attention to social scenes^46,47^. Although the sample size in our study was not large enough to make strong inferences about whether these individual differences transfer to naturalistic settings, they do provide some assurance of measurement reliability at the individual level.

This should enable new work aiming to examine questions relating to individual differences. For example, many lab-based studies have found associations between face-processing ability and face information sampling patterns (e.g.^4,66–71^ for a review see^72^). Relatedly, developmental disorders have been associated with abonomal social attention in laboratory-based studies (e.g. Autism Spectrum Disorders, see^73,74^; Psychopathy, see^75–77^). Whether these associations generalise from lab-based tests to eye movements in real-world environments is an open question.

Second, using automated face detection combined with eye-movement data enabled us to visualise the faces that people fixated on in our study, offering a window into participants’ perceptual experience of faces (see Fig. 3). Limits of the resolution of the video frame meant that we were only able to visualise a small subset of the viewed faces in this study (see Methods), but future methodological and technological development promises to illuminate how social attention shapes a person’s face ‘diet’. Despite the theoretical importance of this exposure in understanding how our perceptual system develops expertise for faces (see^15^) and the continuing development of this expertise in adulthood^78,79^ the amount and quality of perceptual experience people have with faces is currently limited to studies of infants and children (but see^12–14^).

Finally, we believe the present study only scratches the surface of what is possible using this new approach. We expect that the approach will enable new questions to be asked in naturalistic social perception research. Future work could take on ambitious aims, for example, to capture a more complete picture of people's perceptual exposure to faces in their daily lives. Intuitively, this exposure contains rich diversity, such as the familiarity of people we encounter, the contexts and viewing conditions we encounter them in, the nature of our social interactions and the motivations behind them. Characterising the multidimensional nature of this perceptual data and differences in how individuals sample it should offer a critical foundation for the development of theory in this field.

## Methods

All methods were carried out in accordance with relevant guidelines and regulations, were approved by UNSW Human Research Ethics Advisory Panel, and informed consent was obtained from all subjects. Where images of people appear in the figures, all subjects have provided informed consent for publication of identifying information in an online open-access publication.

### Data collection

#### Participants

Thirty-three university students from UNSW Sydney completed the study in return for course credit (9 male, 24 females; Age M = 21.4, SD = 5.4). We did not record participant’s ethnicity. We excluded full data from two participant’s because of corrupt eye-tracking data. In addition, procedural issues meant that data from one segment of the navigation task was deleted for one participant, and face-to-face task data were deleted for three participants. This gave a total of 31 participants in the main navigation task analysis (‘Faces of passersby do not capture attention in live natural settings’), 30 in the individual differences analysis of the navigation task and 28 in the face-to-face interaction analysis.

#### Materials

We used a wearable eye-tracking device to record participants' eye gaze data as they completed the study (Pupil Labs Core:^80^). This device recorded videos of participants’ field of view and eye gaze coordinates. A set of three cameras achieve this recording, a frontal camera facing the environment and two cameras facing the eyes. The resolution of the frontal camera was 1920 × 1080 pixels at 60 frames per second, and the cameras facing the eyes were both of resolution 192 × 192 pixels at 120 frames per second. The wearable eye-tracker was connected via USB to a laptop (Dell XPS 13 7390 2-in-1 placed inside a backpack worn by the participant. We used Pupil Capture to save video and eye gaze data^80^.

After completing the wearable eye-tracking tasks, participants completed a standard measure of unfamiliar face memory ability, the Cambridge Face Memory Test extended version (CMFT+^48^) and a self-report measure of face recognition ability, the Prosopagnosia Index short version (PI-20^49^). This CFMT+ asks for participants to learn and memorise the grayscale faces of 6 caucasian males to be recognised later in 102 three-alternative trials without any time limit. The CFMT+ is a challenging test because the learned faces change in angle of view and image quality in the trials. The PI-20 is composed of 20 questions such as “My face recognition ability is worse than most people”, and participants must rank their responses from “Strongly agree” to “Strongly disagree”.

#### Procedure

We conducted the study during term time when the campus was busy. There were no COVID-19 cases in Sydney at the time and so people were not wearing facemasks. We fitted the mobile eye-tracking device to participants (see Materials above). Participants then completed two tasks while the device recorded their eye gaze: a face-to-face interaction task, where they interacted with the experimenter for a brief period; and a navigation task, where they walked around the UNSW Sydney campus following a circular route.

In the face-to-face interaction task, participants stood in an empty corridor, directly facing the experimenter at a distance of 1.5 m (see Fig. 3A). Participants listened to verbal instructions provided by the experimenter about the navigation task, explaining that this was a naturalistic study and that they should walk through campus as they would on a normal day. The experimenter verbally explained the study before asking participants if they understood and had any questions before begining the task. The experimenter delivered these instructions by reciting a pre-defined script, and participants spent an average of 30 s listening and asking questions about the task. This recording was used in the analysis ‘Fixation patterns during face-to-face interaction associated with face recognition ability’.

Participants then followed the experimenter to a separate room where a detailed map and pictures of the walk were on the wall showing the study route. When participants indicated they were ready to begin, participants exited the room with the experimenter and the Navigation task began. Participants navigated a pre-defined circular route via the main campus thoroughfares passing busy places (e.g. coffee shops, library, food court) through indoor and outdoor settings. Participants were always under the experimenter's supervision, who kept a ~ 2.5 m distance behind participants. When participants arrived at the library, we asked them to stop walking and rest for a minute, which divided the study route into two segments. Segment 1 lasted approximately 12 min on average, and segment 2 approximately 4 min.

When the navigation task was complete, participants removed the wearable eye-tracker before completing the PI-20^49^ and the CFMT+^48^ on a desktop computer. Participants were also asked questions during the debriefing to gauge their awareness of the study’s purpose. Only four participants mentioned attention to people or person perception as a potential research topic.

### Eye gaze data processing

The eye-tracking device collected raw gaze data of participants. We transformed this data into fixations, saccades and blinks using open-source tools provided by the eye-tracking manufacturer (Pupil Capture and Pupil Player, see https://pupil-labs.com/products/core/). Fixations were defined as per default settings, with saccade dispersion of max 1.5° of visual angle, a minimum duration of 80 ms, and a maximum duration of 220 ms. Fixations were output as coordinates labelled to specific pixels on the frontal camera frames. For analysis, we only considered frames with fixations.

Our main methodological advance was to automatically detect the presence of people in the participant's field of view using open-source body and face detection tools (OpenPose:^30^). This tool detects people in video frames and automatically estimates up to 25 landmarks on the body and 70 on the face (if the person is sufficiently close to the viewer). Co-registering fixations with these landmarks enabled us to construct detailed maps of participants' attention to people.

We used two methods to measure participants’ attention to faces and people. In the first method (see Fig. 1A), we registered fixations to the closest detected body or face landmark, considering only landmarks that OpenPose detected with a greater than 60% confidence. We chose a 60% confidence rate because our testing suggested this effectively excluded false positive ‘phantom’ bodies which sometimes briefly appeared in the scene. We calculated the distance between fixation coordinates and landmark coordinates for every frame containing both fixations and landmarks. Where the Euclidean distance between a fixation coordinate and the closest landmark was below a designated threshold we registered a to that landmark. Thresholds varied depending on the spatial resolution of the landmark data being used (navigation task = 70 pixels; face-to-face interaction = 30 pixels). For the purpose of analysis in the navigation task, we clustered 25 landmarks into two categories (face and body; see Fig. 1A) and for the face-to-face interaction task, we clustered 70 facial landmarks into five categories (nose, left/right eye, mouth, and the exterior of the face; see Fig. 3A).

In the second method, we aimed to determine the precise location of fixations in a face to facilitate heatmap analysis in the face-to-face interaction (see Fig. 4). We achieved this by computing the relative position of a given fixation coordinate amongst facial landmarks using Delaunay triangulations^81^ followed by Affine transformations. This way, fixation coordinates that landed within a given computed landmark triangle can be projected on the relative triangle in the standard template. This method enabled us to aggregate fixation data to more precise locations on the face to create a heatmap for each participant during the task.

### Data analysis

#### Navigation task

In the navigation task we registered fixations as being to the head, body, or ‘not-person’ fixations. Head and body fixations were registered when OpenPose had greater than 60% confidence in either head or body regions and when a fixation was detected within 70 pixels of a landmark. These criteria were designed to ensure that we did not underestimate the proportion of fixations to faces and bodies because of random error due to accuracy limits of the eye-tracker (0.6° visual angle see: https://pupil-labs.com/products/core/tech-specs/). Probabilities of fixations to each of these three dynamic regions of interest (dROI) were calculated only for frames where a fixation was recorded. These data were filtered based on OpenPose detection as described in the Results section.

#### Capturing exposure to faces in the wild

First, we collected images of all faces OpenPose detected the full 70 facial landmarks in each participant’s video recording, and sorted these according to whether the participant had fixated on them or not. We then used a face recognition algorithm (ResNet50^82^ trained on the VGGFace2 Database^83^) to find all instances of fixated faces in participants’ recordings. We achieved that by estimating the number of identities in a participant’s video file using K-means clustering and the Elbow method to find the most likely number of identities. This produced sets of images of single identities that had been fixated by participants, and we sorted these into fremes where the face had been fixated and frames where it has not been fixated.

This process provided a set of 1601 images that participants had fixated on and an accompanying set of 4754 images that were of these same face identities but which the participant had not fixated on. We then averaged all the images of each persons face to create an average per face identity and then averaged fixated and non-fixated faces separately to create the images shown in Fig. 5. This was achieved by first morphing face images using Delaunay triangulation with affine transform to align the detected face landmarks on each image to a standard face template. Pixel values from all face images contributing to the average were averaged and the resulting pixel information was morphed to the average face landmark locations.

#### Face-to-Face interaction task

For the face-to-face interaction task, we processed gaze data using landmark and heatmap registration methods. Participant heatmaps were analysed using principal components analysis (PCA) to identify major components (PCs) in the inter-individual variation of heatmaps, returning a set of PCs ranked according to their explained variance (see also^64,66^).The raw input data for the PCA is shown in Supplementary Materials (Fig. S4).

## Supplementary Information

Supplementary Information 1.

Supplementary Video 1.

Supplementary Legends.

## Data Availability

Data supporting analysis are available via https://github.com/UNSWfacelab/Varelaetal_LookingAtFacesInTheWild.
