# Supplementary material for: Looking at faces in the wild
Source: Sci Rep. 2023 Jan 16;13:783. doi: 10.1038/s41598-022-25268-1 (PMC9842722; doi:10.1038/s41598-022-25268-1)
Supplement: Supplementary file 1 — Supplementary Information 1. [file 41598_2022_25268_MOESM1_ESM.pdf]

SUPPLEMENTARY MATERIALS

Looking at faces in the wild

Victor PL Varela[a], Alice Towler[a], Richard Kemp[a], David White[a*]

[a]*University of New South Wales, Australia*

Corresponding Author: Dr David White, david.white@unsw.edu.au

1. Extended analysis for *'Faces of passersby do not capture attention in a live natural setting'*

1.1 Comparing automatic Vs manual coding. To validate the use of OpenPose to detect the presence of a person in a video frame, we randomly sampled 560 frames from participants' navigation task recordings in which a fixation was recorded. Figure S1 shows an example of a frame image. Four naïve volunteers then manually count the number of people in each of the 560 frames. Figure S2 shows the significant positive correlation between the average manual coding values with the automatic values provided by OpenPose ($r(559)= 0.89$, $p<0.001$).

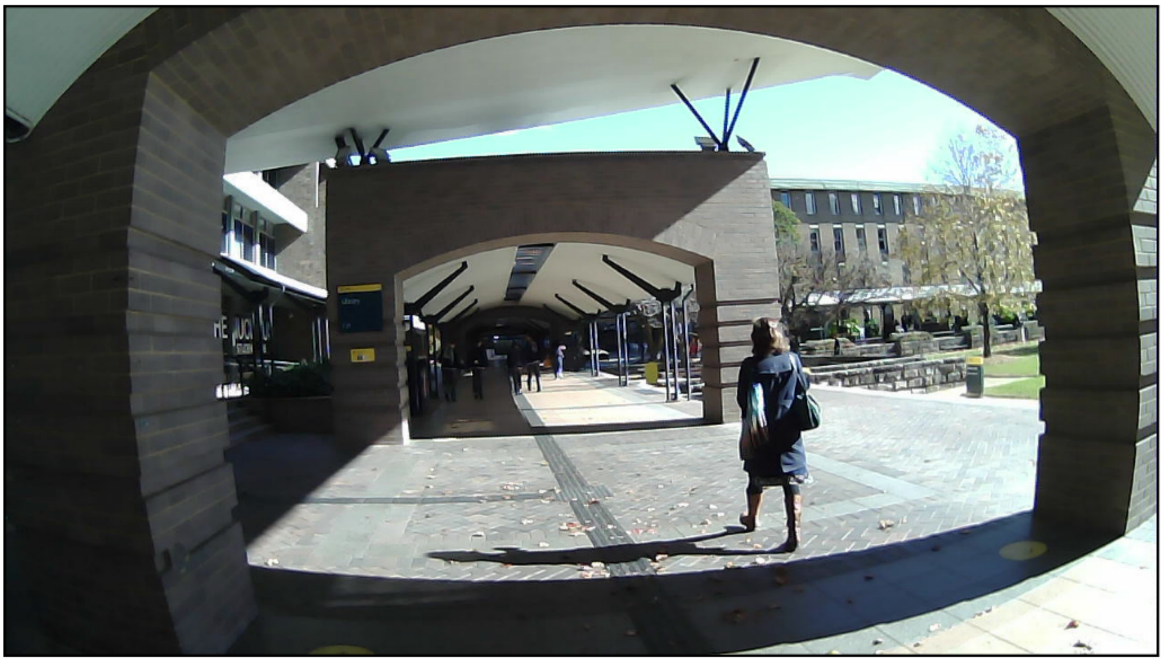

Figure S1. Example of a randomly selected video frame used to validate the OpenPose system.

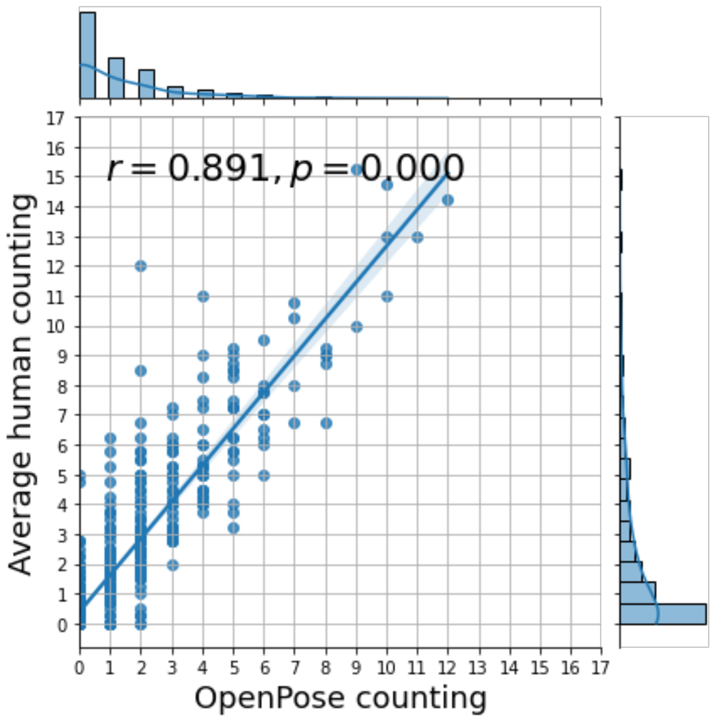

Figure S2. Correlation between human vs algorithm estimates of number of people in the scene

1.2 Size and location of faces in the visual field during navigation and face-to-face interaction tasks. We estimated the size of faces in the participants' visual fields by first measuring degrees of visual angle per pixel of the video frame. We computed this by placing a measurement circle on a wall and extracting video frames where the viewer was standing 50, 100 and 150 cm from the ruler while fixing the circle's centre. We calculated a stable measurement of 0.07° per pixel using basic trigonometry.

Distributions of face size as a function of visual angle are shown separately for the navigation and face-to-face interaction tasks in Figure S3. For the navigation task we computed the size separately for fixated faces (M=2.21°,SD=1.25°,min=0.31°, max=11.45°), and all detected faces (red, M=2.24°,SD=1.28°,min= 0.13°, max= 14.36). Comparing these distributions shows that the faces which were fixated were marginally smaller, t (93208) =-2.22, p=0.026. The average size of faces in the face-to-face task was substantially larger (M= 8.17°, SD=1.17°,min=3.02°, max=11.87°) than in the navigation task.

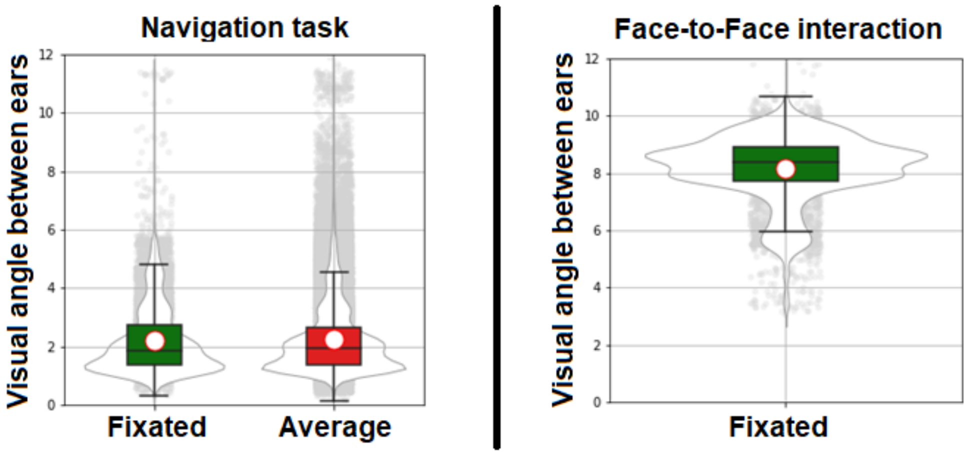

Figure S3. Distributions of detected face size in participants field of view expressed as visual angle. The left panel shows the size of faces detected by OpenPose in the the navigation task, separately for those that were fixated (green) and those that were not (red). The right panel shows the size of faces in the face-to-face interaction task.

For the navigation task we also computed the locations of detected faces in the video file. These locations are visualized in Figure S4, showing that most fixations to faces concentrate at the centre of the participant's field of view. This result could mean that participants turned their heads to look at faces directly. Alternatively, participants may be more likely to look at people's faces when they passed directly in front of them (see Solman, Foulsham & Kingstone, 2017).

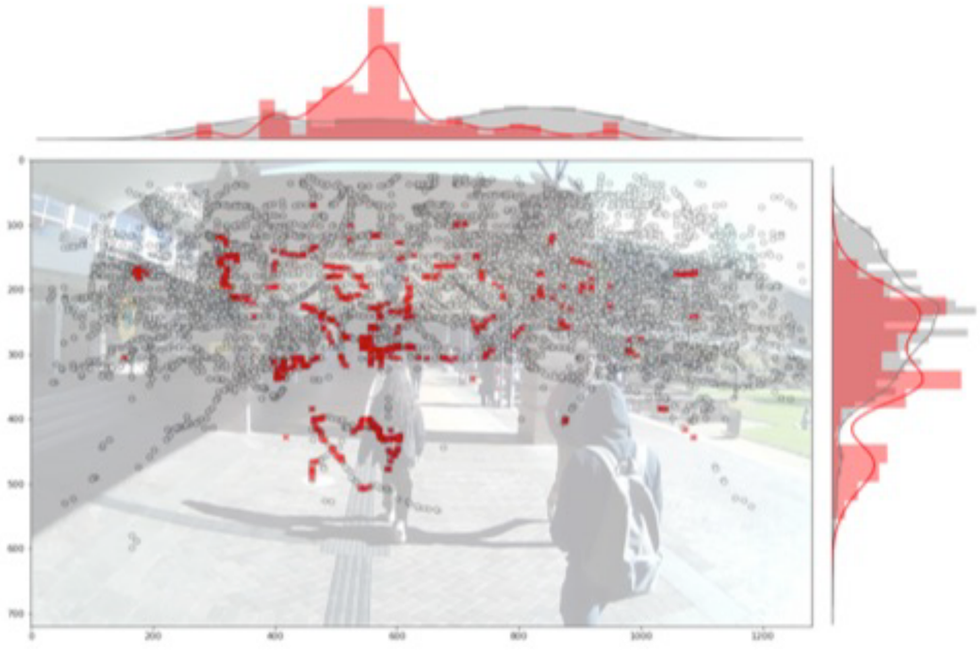

Figure S4. Locations of faces in the field of vision during the navigation task. Spatial distribution of nose location when faces are detected in the video by the algorithm (grey) and for faces that were also fixated by the participant (red). Fixated faces tended to be central in participants' field of view, suggesting that participants mostly directed their attention to people by moving their heads.

1.3 Analysis of face and body fixations as a function of face size. Given the large range of face sizes detected by OpenPose in the navigation task, we also examined whether the size of the face influenced the frequency of fixations to heads and bodies in the scene. Visual inspecton of Figure S5 suggest that the pattern of results was broadly similar for faces subtending smaller than average visual angles and those that were larger than average.

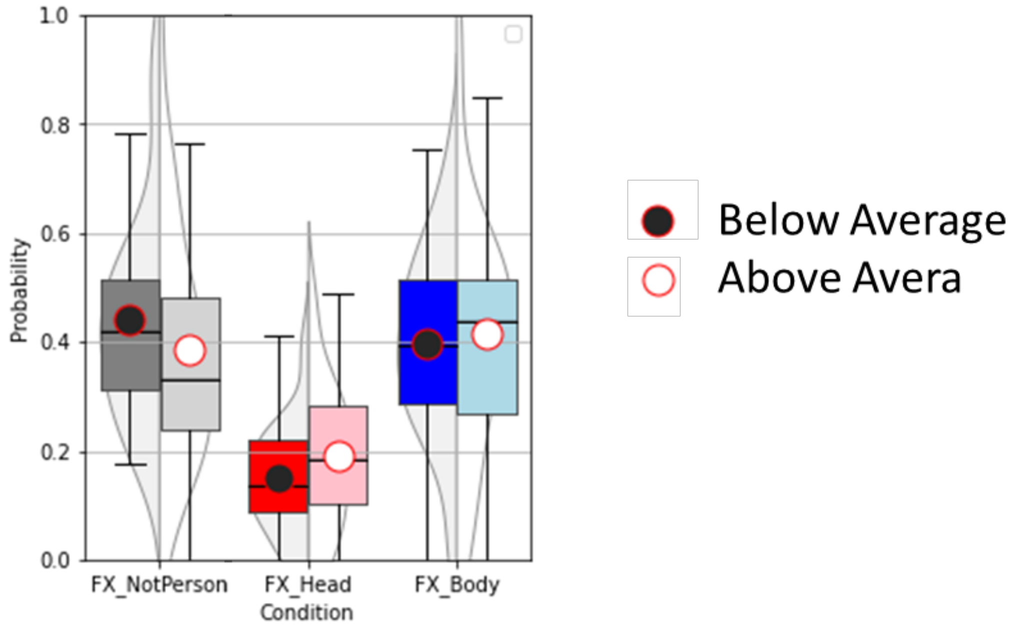

Figure S5. Proportion of fixations to people, heads and bodies as a function of face size. See text for analysis.

We analysed data in Figure S5 using a 2 (below average face size, above average face size) X 2 (Head, Body) repeated measures ANOVA. The main effect of head size was not statistically significant, F (1, 28) = 1.43, p = 0.242, despite numerically larger proportions of fixations to faces and bodies when faces were above average size ($M_{\text{head, below average}}$ = 15.2%, $SD_{\text{head, below average}}$ = 10.0%; $M_{\text{head, above average}}$ = 19.1%, $SD_{\text{head, above average}}$ = 13.4%; $M_{\text{body, below average}}$ = 41.2%, $SD_{\text{body, below average}}$ = 16.0%; $M_{\text{body, above average}}$ = 42.8%, $SD_{\text{body, above average}}$ = 21.4%). The interaction between factors was also non-significant, F (1, 28) = 0.202, p = 0.657.

1.4 Individual participant body maps during the navigation task. We observed high variability in the strategies engaged by participants when looking at people when navigating in the wild. Figure S6 shows the proportion of fixations registered to each landmark separately for each participant in the navigation task.

Participant: P1

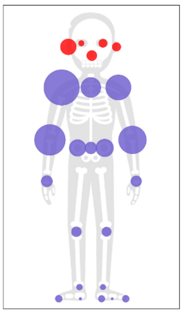

Participant: P2

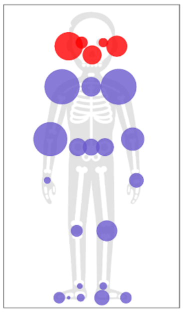

Participant: P3

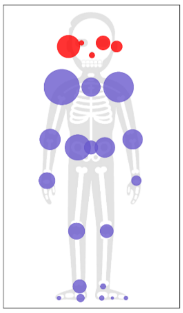

Participant: P4

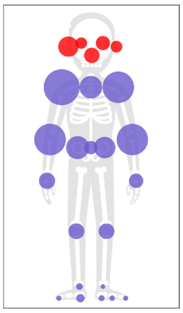

Participant: P5

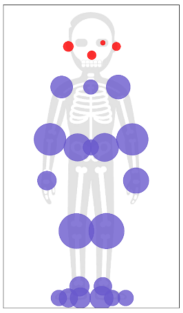

Participant: P6

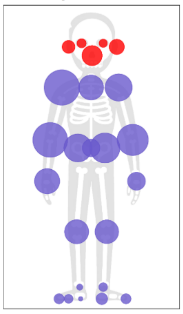

Participant: P7

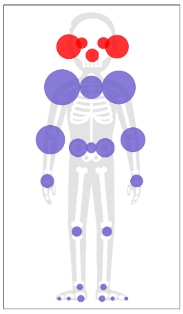

Participant: P8

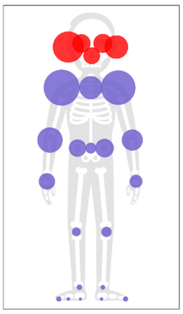

Participant: P9

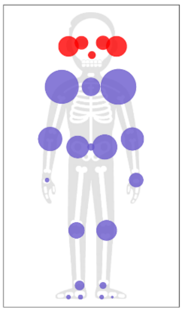

Participant: P10

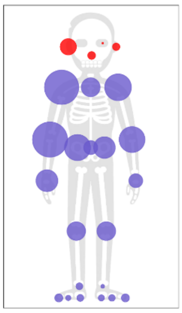

Participant: P11

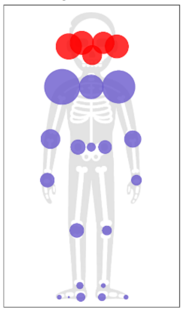

Participant: P12

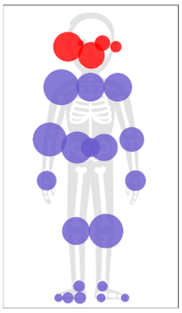

Participant: P13

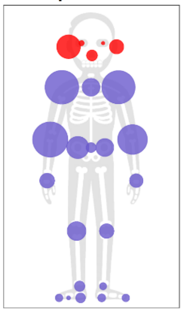

Participant: P14

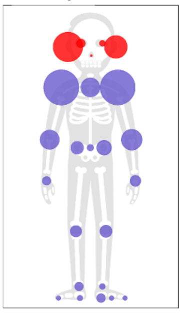

Participant: P15

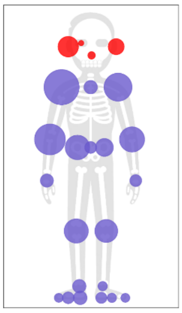

Participant: P16

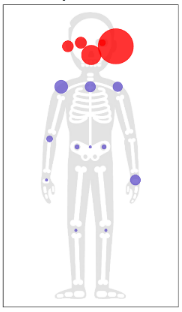

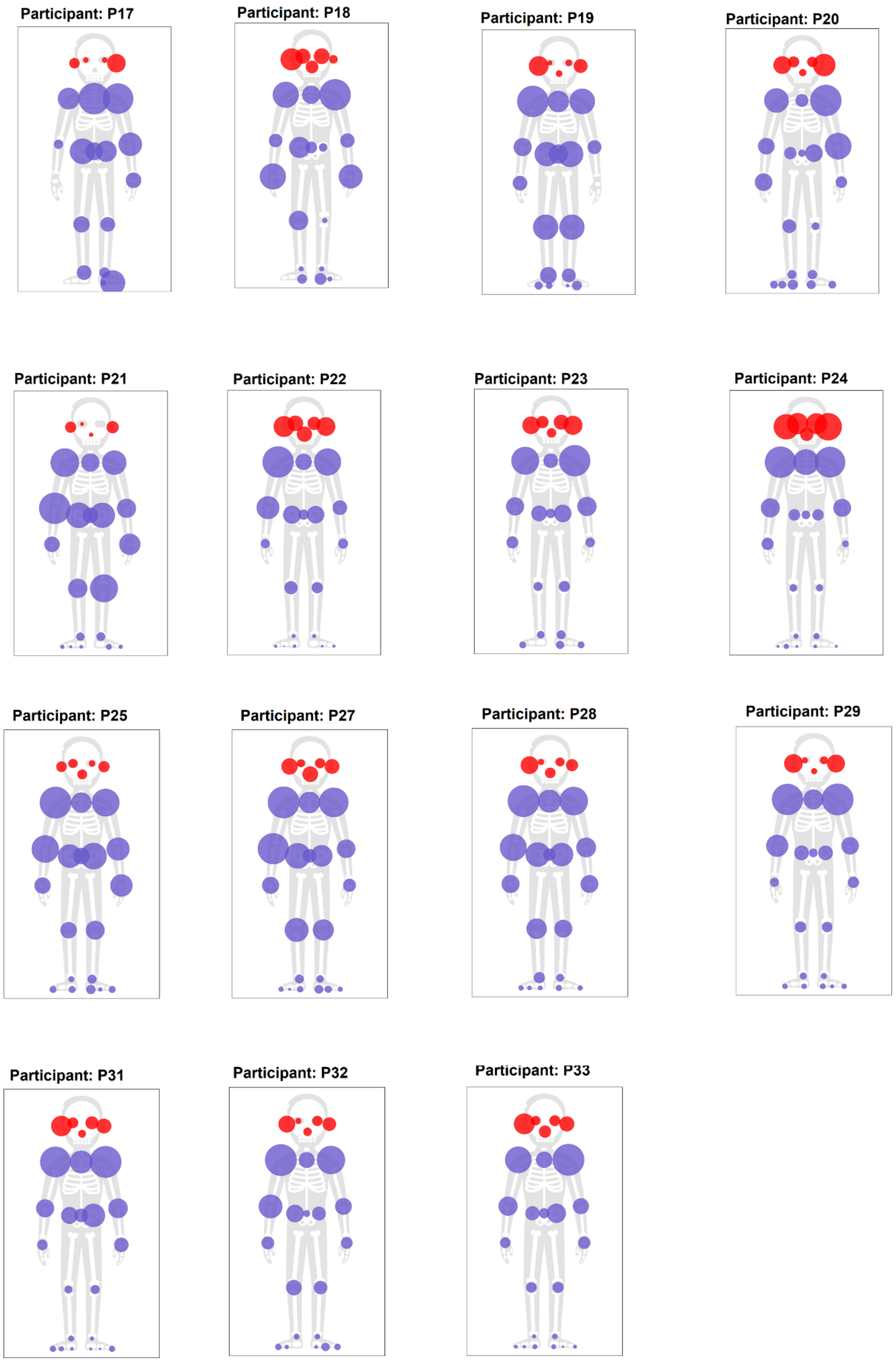

Figure S6. Individual participant data during navigation task. For each participant we show fixations on 25 dROI when viewing people during the navigation task. Face fixations are marked in red and body fixations in blue. The size of the circle for each dROI indicates the proportion of fixations participants made to that location.

2. Extended analysis for '*The effect of frontal Vs profile faces on attention in live natural setting'*

2.1 Manual review of automated full-face Vs averted classification. We conducted a manual review of the ability of our automated method to detect full-face and averted faces. To do this, we extracted 620 frames at random where our method decided that there were only averated faces in the scene and a companion set of 620 frames where full face/s were detected for comparison. To protect the privacy of people in the videos, these images can be requested from the authors for review. Representative examples are provided in Figure S7.

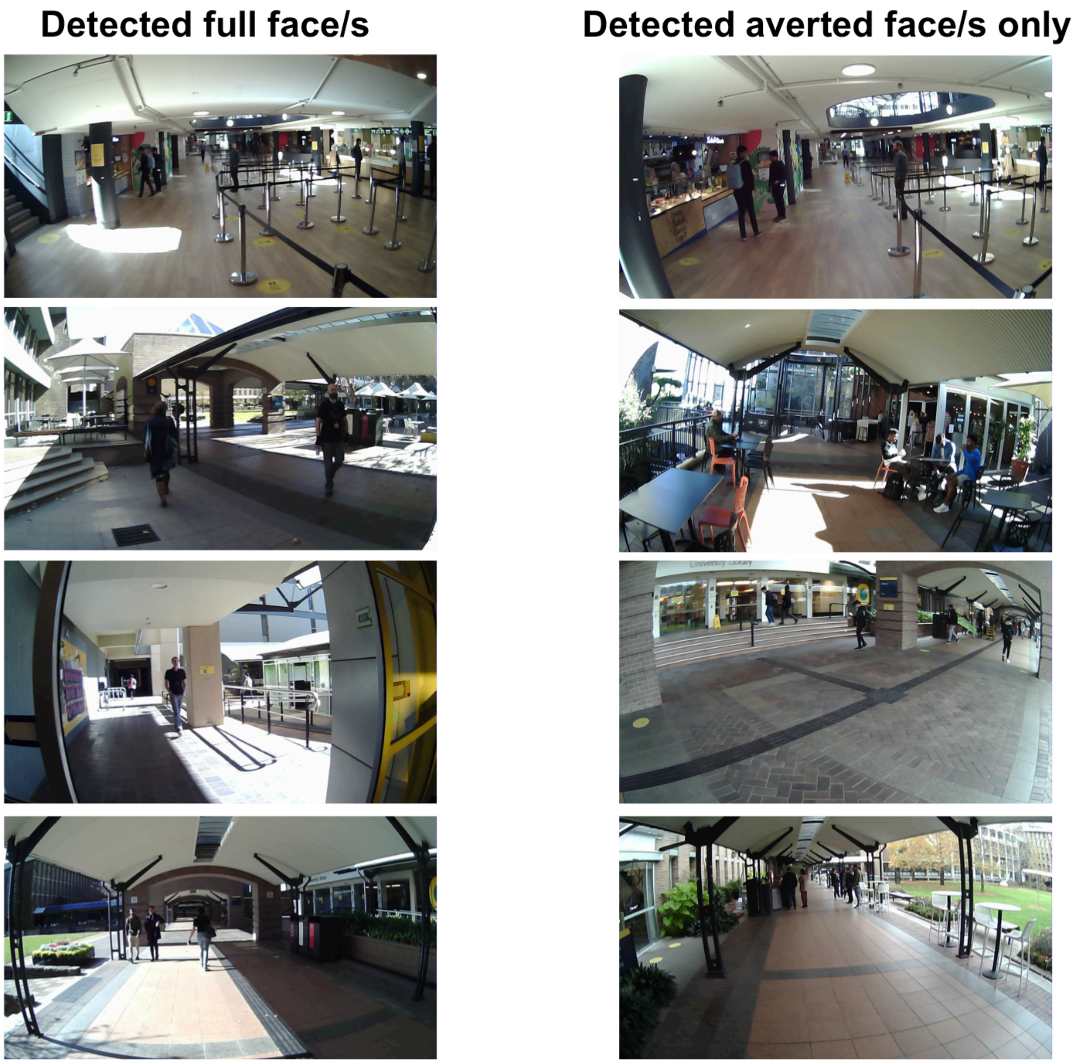

Figure S7. Representative images from the manual review of the full face Vs averted face detection method. We reviewed the method used for analysis in the results section of the main manuscript 'The effect of frontal Vs profile faces on attention in live natural setting' by randomly sampling 620 frames where only full face/s were detected (representative samples on the left) and where only averted face/s were detected (representative samples on the right). Almost all instances were verified as correct classifications, with rare exceptions. For example in the bottom left scene there appears to be one full-face in the crowd in the distance, which has a dark shadow over one half of the face. This face was miscategorised as averted.

The experimenter's review confirmed that in the vast majority of cases, our averted face detection method returned scenes with no full faces. Comparison with the full-face scenes led us to conclude that the method was accurately classifying full-face Vs averted faces. There were some rare cases where the face was classified as averted when experimenters judged it to be full-face because it was occluded by an object/ person (n = 4), by a very dark shadow over half the face (n= 11) or because the face was in the distance (n = 11).

2.2 Full ANOVA analysis. The main manuscript reports that participants were more likely to fixate on people in the navigation task when their faces were in full view. But we found no evidence that faces captured this attention any more than other body regions.

This conclusion was supported by an ANOVA analysis of the data shown in Figure 2 comparing the proportion of fixations to heads and bodies when faces were fully visible in a video frame versus when only partially visible due to head rotation or other occlusions. A 2 (Face type: part face, full face detected) X 3 (Fixation type: Head, Body, Not Person fixations) ANOVA revealed a significant interaction between face and fixation type, $F(2,60) = 9.76$, $p < 0.001$, $\eta^2_p=0.246$. Analysis of simple main effects showed a significant reduction of non-person fixations, $F(1,30) = 12.86$, $p < 0.001$, $\eta^2_p=0.300$, and an increase in both head and body fixations [Head: $F(1,30) = 7.035$, $p = 0.013$, $\eta^2_p=0.190$; Body: $F(1,30) = 6.64$, $p < 0.015$, $\eta^2_p=0.181$].

3.* Extended analysis for '*Fixation patterns during face-to-face interaction'

3.1 Individual participant fixation maps during the face-to-face interaction task

We observed high variability in the fixation patterns shown by participants when engaging in a conversation with the experimenter. We filtered participants' recordings to analyse only fixation frames that contained the experimenter's face looking straight at the participant by using only frames where nose landmarks were detected by OpenPose (Cao et al., 2019). The landmark registration method (see the main manuscript) detected 70 possible dynamic regions of interest (dROI) participants attended. Figure S8 shows individual participant gaze patterns registered to facial landmarks.

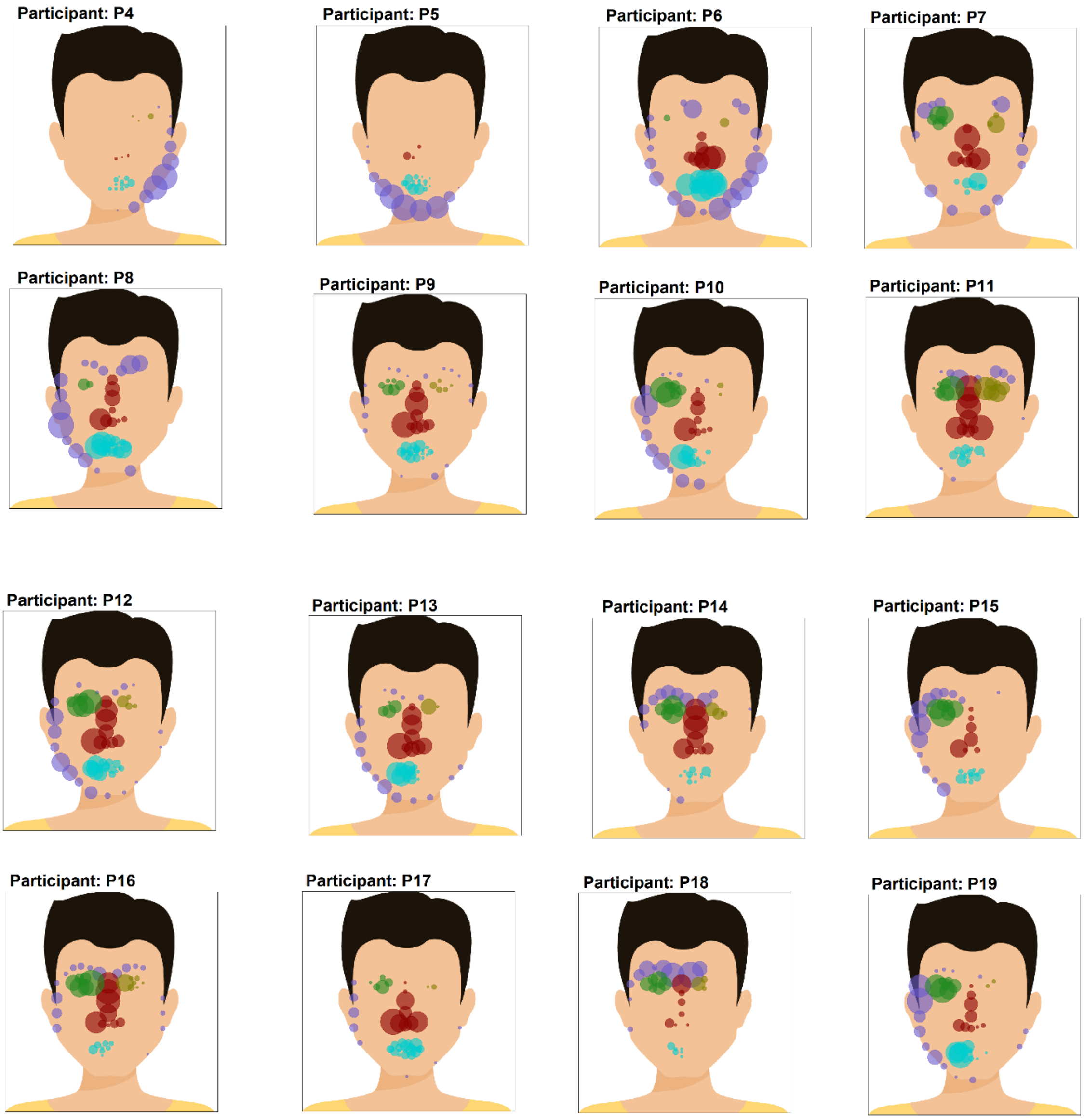
Participant: P4
Participant: P5
Participant: P6
Participant: P7
Participant: P8
Participant: P9
Participant: P10
Participant: P11
Participant: P12
Participant: P13
Participant: P14
Participant: P15
Participant: P16
Participant: P17
Participant: P18
Participant: P19

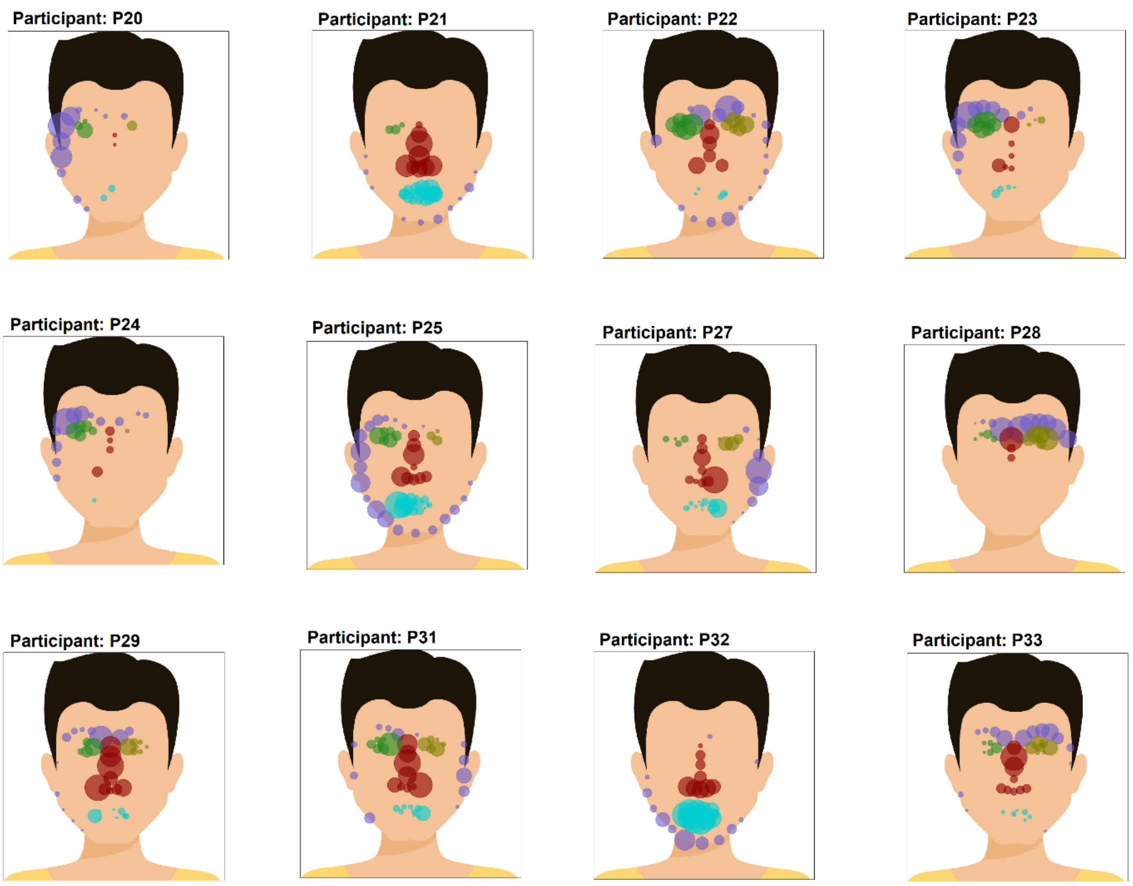

Figure S8. Individual participant fixation data during face-to-face interaction task. We show fixations to the 70 dROI identified by OpenPose while participants attended to the experimenter during the face-to-face interaction task. The colours delimit facial regions for this visualisation, and the size of the circles at each dROI indicates the number of fixations at that landmark.

3.2 Individual participant heatmaps during the face-to-face interaction task.

The landmark registration in Figure S8 is constrained to record 70 possible dROI positions on a face. However, the heatmap registration method allows more fine-grained analysis because it uses the relation between these landmarks to determine the exact location of where a fixation landed on a face (see main text). Figure S9 shows individual heatmaps for each participant as they focused on the experimenter's faceduring the face-to-face task. Comparing the patterns in Figures S8 and S9 shows close correspondence between the maps generated by these two methods, but also points to advantages of using triangulation of spatial location. For example, when analysed using landmark registration, P25 appears to spread fixations around the contour of the face, but this is revealed as a more focal pattern on the mouth area when using triangulation.

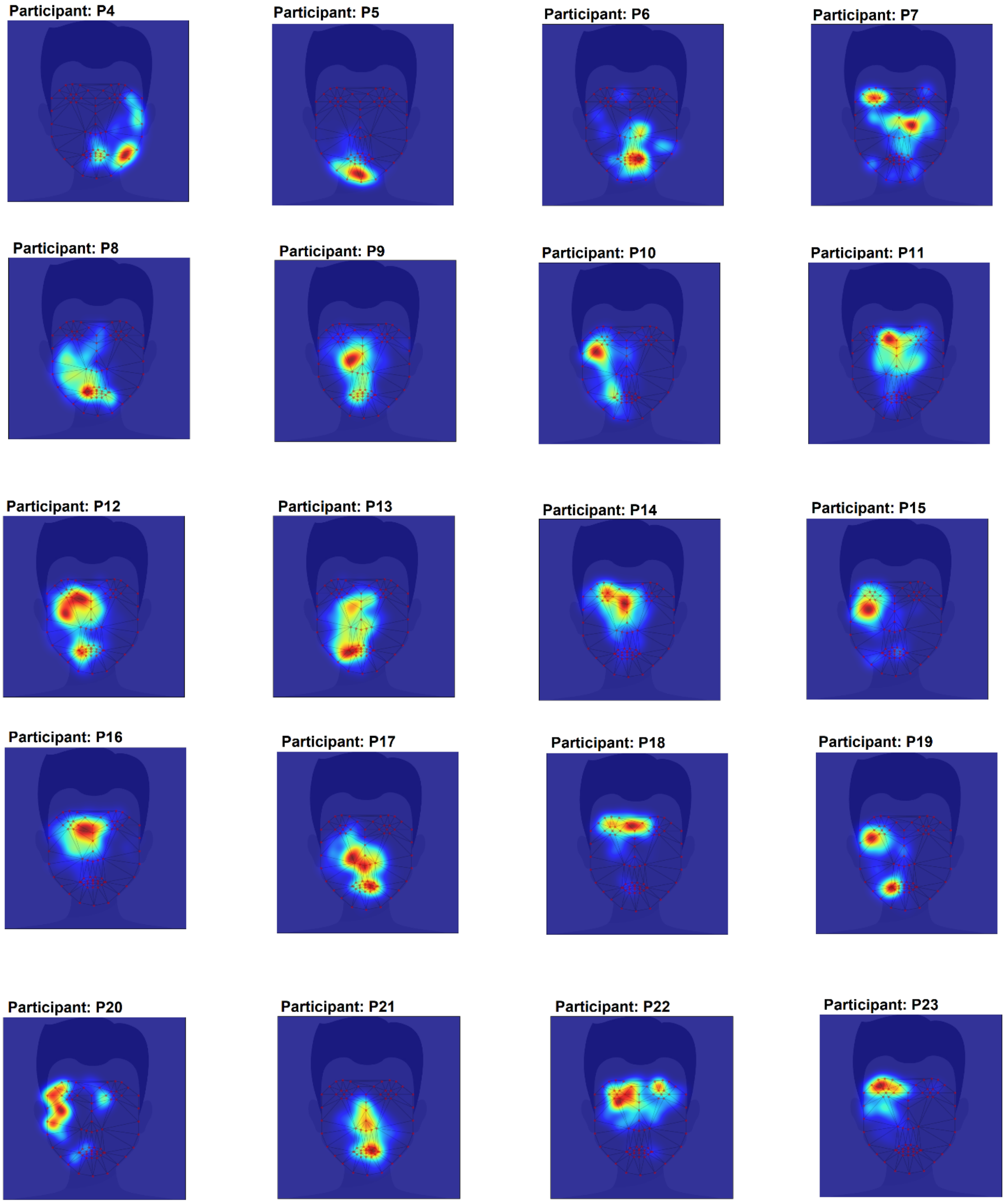
Participant: P4
Participant: P5
Participant: P6
Participant: P7
Participant: P8
Participant: P9
Participant: P10
Participant: P11
Participant: P12
Participant: P13
Participant: P14
Participant: P15
Participant: P16
Participant: P17
Participant: P18
Participant: P19
Participant: P20
Participant: P21
Participant: P22
Participant: P23

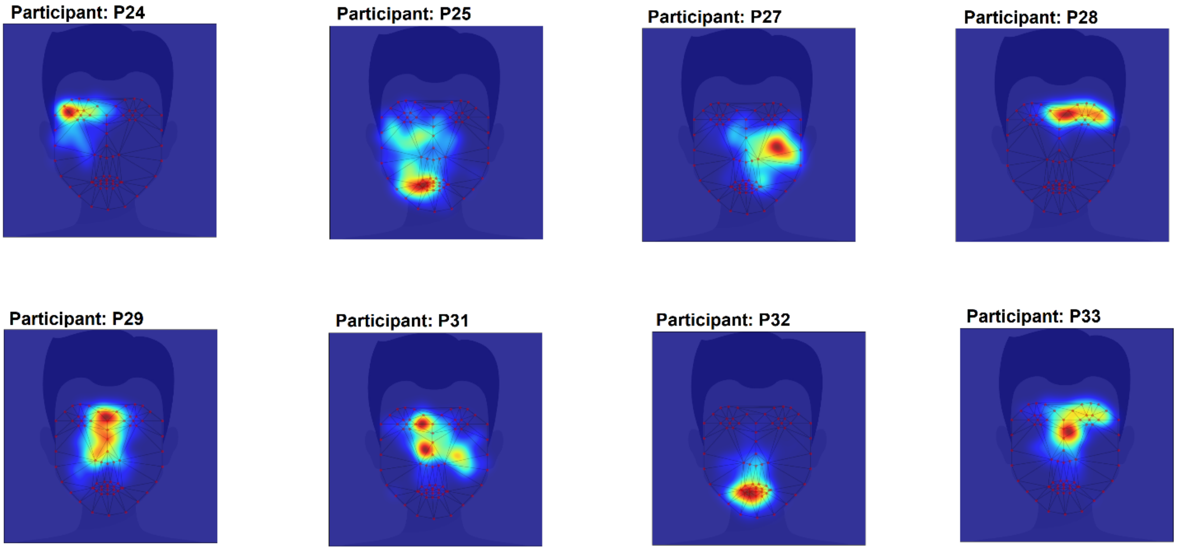

Figure S9. Individual participantheatmap data during face-to-face interaction task. See main manuscript for details.

4. Extended analysis for 'Individual differences in naturalistic social attention'

4.1 Stability of individual differences across two segments of the navigation task. In the main text we report a significant correlation between fixations to people in the two segments of the navigation route, which is visualised below as a scatterplot in Figure S10.

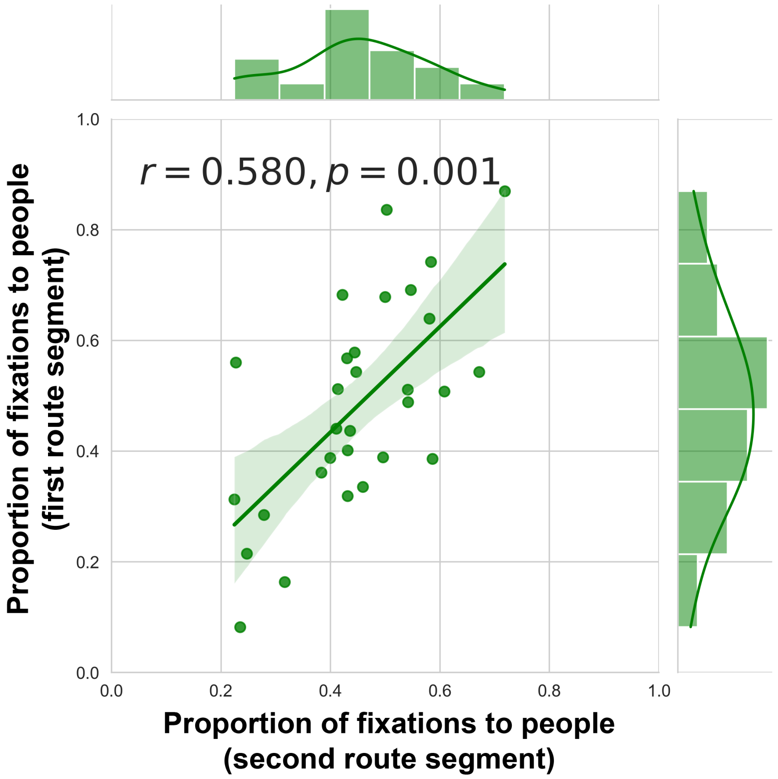

Figure S10. Stable individual differences in proportions of fixations to people across two segments of the navigation route. Correlation between the proportion of fixations made by individual participants to people in route second segment as a function of the first segment.

To carry out the analysis of residuals reported in the main paper we first calculated the linear regression model predicting the probability of fixating people (head and body) as a function of the average number of people detected in video frames, separately for the two route segments (First and Second). Figure S11A shows these scatterplots and linear models, which allowed us to calculate a residual value for each participant from the regression lines separately for each route segment. Figure S11B shows the correlation between these residuals for the two route segments (Spearman's rho(29)= 0.532, p= 0.002), indicating that some participants tend to fixate more on people than others, regardless of the number of people they encountered on the walk.

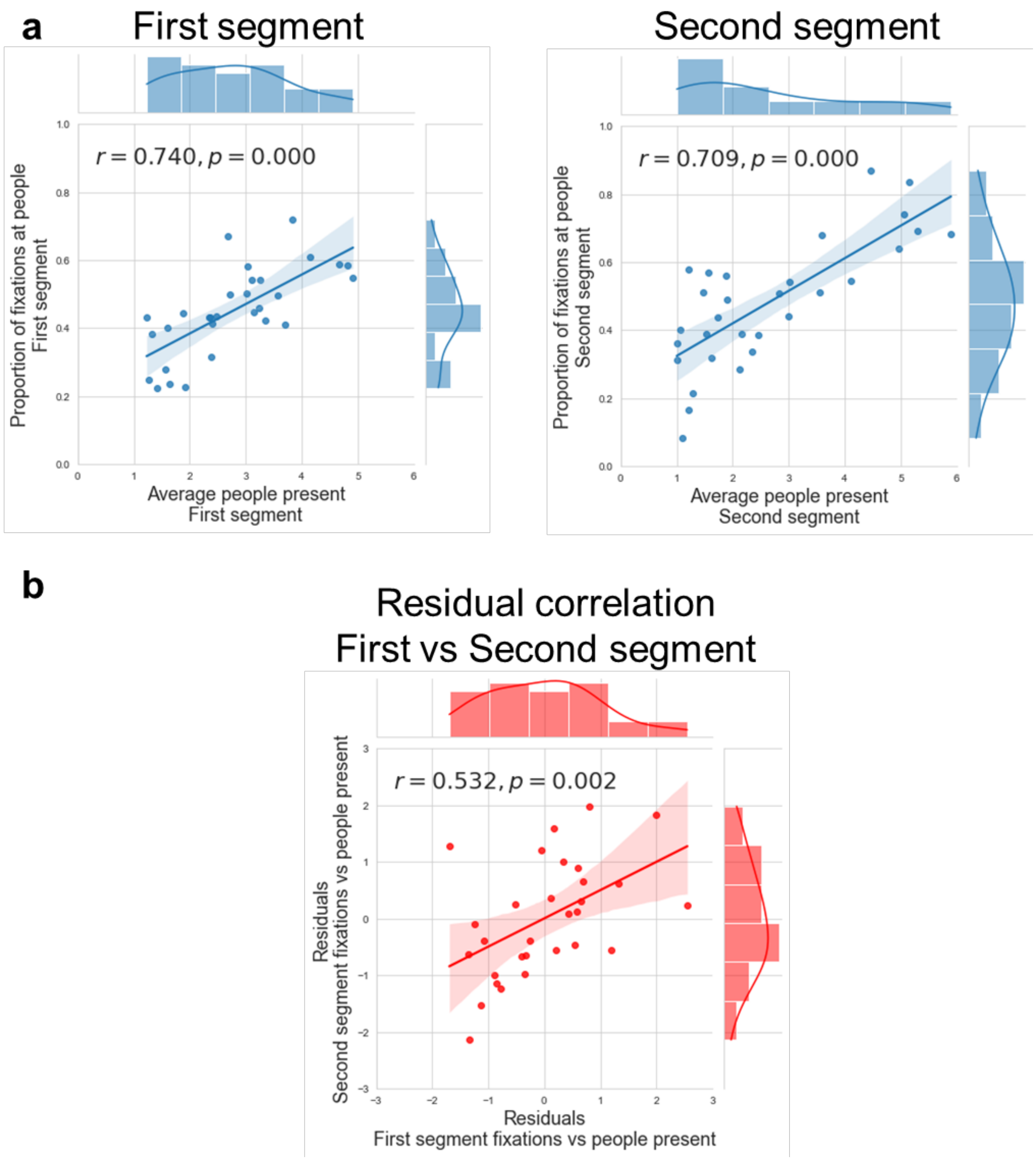

Figure S11. Individual differences analysis of residuals. Panel A shows the proportion of fixations to people as a function of the average number of people present per video frame for each route segment. Panel B shows the correlation between the residuals found in panel A.

4.2 Association between attention to people in the navigation task and face identity processing ability. Having found reliable individual differences in participants' tendency to fixate on people in their environment, we asked whether these differences were related to face identity processing ability measures. We found no significant correlation between the proportion of fixations to people and their score on an objective (CFMT+: Spearman's rho =-0.166, p=0.371, CI=[-0.48, 0.54]) or self-report measure of face recognition ability (PI-20: Spearman's rho =0.117,

p=0.529,CI=[-0.44, 0.23]) (see Methods – Materials). Because previous work has shown that people with high levels of face recognition ability focus more on faces in natural scenes (Bobak et al., 2017), we also repeated this analysis by examining the proportion of fixations to faces only, but again we found no association (CFMT+: Spearman's rho= -0.073, p= 0.696, CI= [-.41, 0.27]; PI-20: Spearman's rho= -0.023, p= 0.901, CI= [-0.36, 0.32]). Finally, we repeated this analysis but correlating PI-20 and CFMT+ scores with the residuals of correlations shown in Figure S11A and again found no association (rhos < .050).

4.3 Association between fixation patterns to faces in face-to-face and face identity processing ability. Previous work shows substantial variation in the gaze patterns of individual participants (e.g. Mehoudar, Arizpe, Baker, & Yovel, 2014; Arizpe, Walsh, Yovel, & Baker, 2017; Dunn et al., 2022). Following our recent eye-tracking work using static eye trackers (Varela et al., 2018; Dunn et al., 2022), we used principal component analysis (PCA) to explore the underlying dimensions of this individual variation in our participant heatmaps (see Methods – Data analysis). Figure S12A visualizes the first five principal components in participants' average heatmaps during the face-to-face task. Visual inspection and follow up analysis showed that the main source of variation (PC1) captured a shift in participants that focussed on the eye region to those that attended to the mouth (Correlation between PC1 and mouth landmark fixation count (n = 28): Spearman's rho= 0.897, p< 0.001, CI= [0.79, 0.95]; Left eye: Spearman's rho= -0.724, p< 0.001,CI= [-0.85, -0.49]; Right eye: Spearman's rho= -0.513, p= 0.005,CI= [0.18, 0.73]).

In an exploratory analysis, we also found weak evidence that people with higher face recognition ability made more fixations on the eyes in face-to-face interaction. We measured the association between participants' PC loadings and face recognition ability measured by score on the Cambridge Face Memory Test extended version (CFMT+; see Russell et al. 2009). PC1 showed a significant correlation with face recognition performance (Spearman’s rho= -0.44, p= 0.019, CI= [-0.69, -0.09]), whereby higher CFMT+ scores were associated with more focus on the eye region (see Figure S12B). There were no significant correlations with face recognition performance for the other components (PC2 – PC5). The full correlation statistics are shown in Table S1, showing unadjusted p-values.

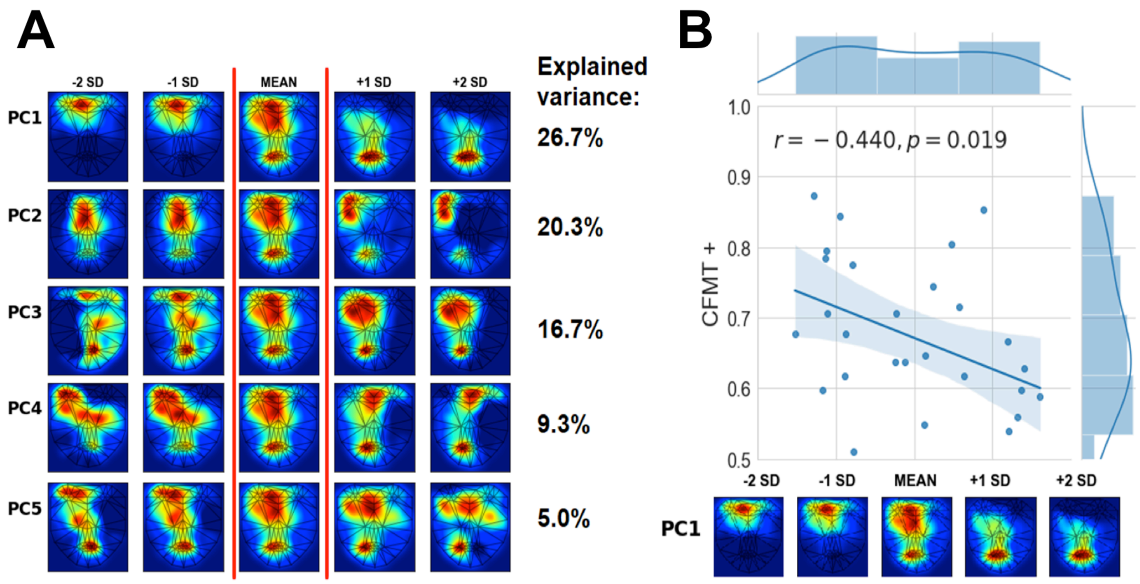

Figure S12. Principal component analysis of fixation heatmaps in the face-to-face interaction. (A) We analysed participant heatmaps using principal component analysis to explore the primary sources of variation in participants' viewing patterns. Here, we show its interaction with the average heatmap in the five first principal components, which accounted for ~78% of the variance. The first principal component explained ~27% of the variance in viewing patterns and captured individual differences in focus on the eye versus mouth regions. (B) Fixations to eyes were associated with higher levels of face recognition ability as measured by the CFMT+.

While some screen-based studies also report increased eye-region fixations for people with higher face identity processing ability (Wilcockson, Burns, Xia, Tree, & Crawford, 2020; see also Tardif et al., 2019), others report no association (e.g. Arzipe et al., 2017), and others find the opposite result (e.g. Dunn et al., 2022). Although this inconsistency might reflect the small sample sizes typical of eye-tracking individual difference studies, it may also point to the importance of situational context, viewer's goals and motivation in the information sampled from a face. This can be extended in future work by recruiting larger participant groups to examine the relationship between face processing, social attention and social cognition in more diverse settings and situations.

		CFMTp		PI20
PI20	Spearman's rho	-0.175		—
	p-value	0.373		—
PC1	Spearman's rho	-0.44	*	0.343
	p-value	0.019		0.074
PC2	Spearman's rho	-0.124		-0.087
	p-value	0.531		0.659
PC3	Spearman's rho	0.202		-0.294
	p-value	0.302		0.129
PC4	Spearman's rho	0.23		-0.115
	p-value	0.24		0.559
PC5	Spearman's rho	0.126		0.098
	p-value	0.524		0.619
FX_Nose	Spearman's rho	0.088		0.032
	p-value	0.656		0.871
FX_LeftEye	Spearman's rho	0.252		-0.073
	p-value	0.197		0.712
FX_RightEye	Spearman's rho	0.397	*	-0.333
	p-value	0.036		0.083
FX_Mouth	Spearman's rho	-0.271		0.339
	p-value	0.163		0.077
FX_Exterior	Spearman's rho	0.009		-0.039
	p-value	0.964		0.842
FX_OtherAreas	Spearman's rho	-0.11		0.092
	p-value	0.579		0.64

Note. * p < .05, ** p < .01, *** p < .001

Table S1. Correlation between face identification measures and eye fixation

References

Arizpe, J., Walsh, V., Yovel, G., & Baker, C. I. (2017). The categories, frequencies, and stability of idiosyncratic eye-movement patterns to faces. *Vision Research*, *141*, 191-203.

Cao, Z., Hidalgo, G., Simon, T., Wei, S. E., & Sheikh, Y. (2019). OpenPose: realtime multi-person 2D pose estimation using Part Affinity Fields. *IEEE transactions on pattern analysis and machine intelligence*, *43*(1), 172-186.

Dunn, J. D., Varela, V. P., Nicholls, V. I., Papinutto, M., White, D., & Miellet, S. (2022). Face-Information Sampling in Super-Recognizers. *Psychological Science*, *33*(9), 1615-1630.

Mehoudar, E., Arizpe, J., Baker, C. I., & Yovel, G. (2014). Faces in the eye of the beholder: Unique and stable eye scanning patterns of individual observers. *Journal of vision*, *14*(7), 6-6.

Solman, G. J., Foulsham, T., & Kingstone, A. (2017). Eye and head movements are complementary in visual selection. Royal Society Open Science, 4(1), 160569.

Tardif, J., Morin Duchesne, X., Cohan, S., Royer, J., Blais, C., Fiset, D., ... & Gosselin, F. (2019). Use of face information varies systematically from developmental prosopagnosics to super-recognizers. *Psychological science*, *30*(2), 300-308.

Varela, V. P., Ribeiro, E., Orona, P. A., & Thomaz, C. E. (2018, October). Eye movements and human face perception: An holistic analysis and proficiency classification based on frontal 2D face images. In *Anais do XV Encontro Nacional de Inteligência Artificial e Computacional* (pp. 48-57). SBC.

Wilcockson, T. D., Burns, E. J., Xia, B., Tree, J., & Crawford, T. J. (2020). Atypically heterogeneous vertical first fixations to faces in a case series of people with developmental prosopagnosia. Visual Cognition, 28(4), 311-323.
