# Supplementary material for: Looking at faces in the wild
Source: Sci Rep. 2023 Jan 16;13:783. doi: 10.1038/s41598-022-25268-1 (PMC9842722; doi:10.1038/s41598-022-25268-1)
Supplement: Supplementary file 3 — Supplementary Legends. [file 41598_2022_25268_MOESM3_ESM.docx]

**Supplementary Video.** Recording from a wearable eye-tracker showing face and body detection by OpenPose [30] on the left and participant fixations calculated by Pupil Capture [78] on the right. In the middle, we show how we compute proportions of fixations to body and face regions as data is accumulated over the course of the face-to-face interaction.
